# Linkage mapping, molecular cloning and functional analysis of soybean gene *Fg3* encoding flavonol 3-*O*-glucoside/galactoside (1 → 2) glucosyltransferase

**DOI:** 10.1186/s12870-015-0504-7

**Published:** 2015-05-23

**Authors:** Shaokang Di, Fan Yan, Felipe Rojas Rodas, Tito O Rodriguez, Yoshinori Murai, Tsukasa Iwashina, Satoko Sugawara, Tetsuya Mori, Ryo Nakabayashi, Keiko Yonekura-Sakakibara, Kazuki Saito, Ryoji Takahashi

**Affiliations:** Graduate School of Life and Environmental Sciences, University of Tsukuba, Tsukuba, Ibaraki 305-8518 Japan; Department of Botany, National Museum of Nature and Science, Tsukuba, Ibaraki 305-0005 Japan; RIKEN Center for Sustainable Resource Science, Yokohama, Kanagawa 230-0045 Japan; National Institute of Crop Science, Tsukuba, Ibaraki 305-8518 Japan; Current address: Universidad Católica de Oriente, Rionegro-Antioquia, Colombia

**Keywords:** *Fg3* gene, Flavonol glycoside, Flavonol 3-*O*-glucoside/galactoside (1 → 2) glucosyltransferase, *Glycine max*, Soybean

## Abstract

**Background:**

Flavonol glycosides (FGs) are major components of soybean leaves and there are substantial differences in FG composition among genotypes. The first objective of this study was to identify genes responsible for FG biosynthesis and to locate them in the soybean genome. The second objective was to clone the candidate genes and to verify their function. Recombinant inbred lines (RILs) were developed from a cross between cultivars Nezumisaya and Harosoy.

**Results:**

HPLC comparison with authentic samples suggested that FGs having glucose at the 2″-position of glucose or galactose that is bound to the 3-position of kaempferol were present in Nezumisaya, whereas FGs of Harosoy were devoid of 2″-glucose. Conversely, FGs having glucose at the 6″-position of glucose or galactose that is bound to the 3-position of kaempferol were present in Harosoy, whereas these FGs were absent in Nezumisaya. Genetic analysis suggested that two genes control the pattern of attachment of these sugar moieties in FGs. One of the genes may be responsible for attachment of glucose to the 2″-position, probably encoding for a flavonol 3-*O*-glucoside/galactoside (1 → 2) glucosyltransferase. Nezumisaya may have a dominant whereas Harosoy may have a recessive allele of the gene. Based on SSR analysis, linkage mapping and genome database survey, we cloned a candidate gene designated as *GmF3G2″Gt* in the molecular linkage group C2 (chromosome 6). The open reading frame of *GmF3G2″Gt* is 1380 bp long encoding 459 amino acids with four amino acid substitutions among the cultivars. The *GmF3G2″Gt* recombinant protein converted kaempferol 3-*O*-glucoside to kaempferol 3-*O*-sophoroside. GmF3G2″Gt of Nezumisaya showed a broad activity for kaempferol/quercetin 3-*O*-glucoside/galactoside derivatives but it did not glucosylate kaempferol 3-*O*-rhamnosyl-(1 → 4)-[rhamnosyl-(1 → 6)-glucoside] and 3-*O*-rhamnosyl-(1 → 4)-[glucosyl-(1 → 6)-glucoside].

**Conclusion:**

*GmF3G2″Gt* encodes a flavonol 3-*O*-glucoside/galactoside (1 → 2) glucosyltransferase and corresponds to the *Fg3* gene. *GmF3G2″Gt* was designated as UGT79B30 by the UGT Nomenclature Committee. Based on substrate specificity of *GmF3G2″Gt*, 2″-glucosylation of flavonol 3-*O*-glycoside may be irreconcilable with 4″-glycosylation in soybean leaves.

**Electronic supplementary material:**

The online version of this article (doi:10.1186/s12870-015-0504-7) contains supplementary material, which is available to authorized users.

## Background

Leaves of soybean (*Glycine max* (L.) Merr.) contain a variety of flavonol glycosides (FGs) that are derivatives of quercetin and kaempferol [1]. Buzzell and Buttery [2] proposed four flavonol glycoside alleles, viz., *Fg1* (β(1–6)-glucoside present), *Fg2* (α(1–6)-rhamnoside present), *Fg3* (β(1–2)-glucoside present), and *Fg4* (α(1–2)-rhamnoside present). These alleles are defined by their ability to bind glucose or rhamnose at either position 2″ or 6″ to the glucose moiety that is bound to the 3-position of flavonols. Later, Buzzell and Buttery [3] reported a new allele of the *Fg2* locus, resulting in a series of alleles, *Fg2-a*, *Fg2-b* and *fg2. Fg3* and *Fg4* are linked with a recombination frequency of 12% in the molecular linkage group C2 (chromosome 6) [4]. Plants with the *Fg1Fg3* alleles have a lower rate of photosynthesis, lower leaf chlorophyll concentration, lower leaf weight, and lower seed yield [5]. Further, *Fg1* and *Fg3* control waviness of leaf margins in soybean [6].

Glycosyltransferases (GTs) catalyze the transfer of sugar moieties from activated donor molecules to specific acceptor molecules, forming glycosidic bonds [7]. GTs are classified into at least 96 families (GT1-GT96, http://www.cazy.org/GlycosylTransferases.html). The family 1 glycosyltransferase, referred to as UDP glycosyltransferases (UGTs), comprise the largest group in plants. UGTs catalyze the transfer of a glycosyl moiety from UDP sugars to a wide range of acceptor molecules including flavonoids [8].

Rojas Rodas et al. [9] performed genetic analysis using RILs derived from a cross between Koganejiro and Kitakomachi which are soybean cultivars with gray pubescence. FGs of Koganejiro had rhamnose at the 6″-position of glucose or galactose that is bound to the 3-position of kaempferol, whereas FGs of Kitakomachi were devoid of rhamnose. The presence of 6″-rhamnose was controlled by a single gene. They cloned a candidate gene, *GmF3G6″Rt*, in the molecular linkage group O (chromosome 10). The recombinant *GmF3G6″Rt* protein converted UDP-rhamnose and kaempferol 3-*O*-glucoside or kaempferol 3-*O*-galactoside to kaempferol 3-*O*-rutinoside or kaempferol 3-*O*-robinobioside, proving that *GmF3G6″Rt* encodes a flavonol 3-*O*-glucoside/galactoside (1 → 6) rhamnosyltransferase and corresponds to the *Fg2* gene. Thus, either glucose or galactose was attached to the 3-position of kaempferol partially contradicting Buttery and Buzzell [10] who reported that only glucose was attached to the 3-position. In addition, FGs having rhamnose at the 4″-position of 3-*O*-galactose, kaempferol 3-*O*-rhamnosyl-(1 → 4)-[rhamnosyl-(1 → 6)-galactoside] and kaempferol 3-*O*-rhamnosyl-(1 **→** 4)-[glucosyl-(1 **→** 6)-galactoside] have been identified in the leaves of soybean [9,11], suggesting the existence of flavonol 3-*O*-galactoside (1 → 4) rhamnosyltransferase in soybean. Hence, the genetic control of FG biosynthesis proposed by Buttery and Buzzell [10] should be revised. Overall, glucose or galactose is attached to the 3-position of kaempferol or quercetin in the biosynthesis of FGs in soybean leaves. Glucose can be attached to the 2″- or 6″-positions of glucose or galactose whereas rhamnose can be attached to the 2″-, 4″- or 6″-positions, resulting in a wide variety of FGs [9].

Preliminary experiments suggested that a Japanese landrace, Nezumisaya, and a Canadian cultivar Harosoy, both with gray pubescence, had different FG compositions in leaves (T. Iwashina et al., unpublished results, 2010). The first objective of this study was to identify genes for FG biosynthesis and locate them in the soybean genome using RILs derived from a cross between Nezumisaya and Harosoy. The second objective was to clone and verify their function.

## Methods

### Plant materials

Nezumisaya with yellow hilum, yellow seed coats, gray pubescence and purple-blue flowers (*IIttW1W1w2w2w3w3W4W4WmWmWpWp*) was crossed with Harosoy with yellow hilum, yellow seed coats, gray pubescence and purple flowers (*IIttW1W1W2W2w3w3W4W4WmWmWpWp*). Flowers of Nezumisaya were emasculated one day before opening and fertilized with pollen from Harosoy in 2004. Hybridity of F_1_ plants was ascertained based on the presence of purple flowers. A total of 120 RILs of F_6_ generation were developed without any selection by the single-seed descent method. Seeds were planted at the National Institute of Crop Science, Tsukuba, Japan (36°06′N, 140°05′E) on June 9, 2011. N, P, and K were applied at 3.0, 4.4, and 8.3 g m^−2^, respectively. Plants were individually planted 10 cm apart within rows that were spaced 70 cm apart. On average, nine plants were grown for each parent and RIL.

### Extraction of FGs

A total of 94 RILs was randomly selected and used for analysis because PCR reaction plates and electrophoresis apparatus were designed for multiples of 96 samples (94 RILs and 2 parents). Trifoliolate leaves were collected in bulk from 4 plants from the parents and the RILs at R6 stage [12]. Sampling of leaves and preparation of HPLC samples were performed as previously described [9]. Ten μl from each sample was subjected to high performance liquid chromatography (HPLC) analysis.

### HPLC and genetic analyses

Quantitative HPLC separation of the extracts was performed with the Agilent 1100 HPLC System (Agilent Technologies) using L-column 2 ODS [I.D. 6.0 × 150 mm (Chemicals Evaluation and Research Institute)] at a flow rate of 1.0 ml/min, detection wavelengths from 190–700 nm and phosphoric acid/acetonitrile/H_2_O (0.2:18:82) as eluent. FGs were detected at 350 nm wavelength and their corresponding amounts were estimated from the pertinent peak areas in the HPLC chromatogram. The genetic model for FG composition was hypothesized based on the HPLC chromatogram of the parents and the RILs, and the chemical structure of the pertinent peaks. Likelihood of the genetic model was estimated by Chi-square test.

### SSR analysis and linkage mapping

Genomic DNA of the parents and the four plants from each of the RILs used for FG analysis were isolated from trifoliolate leaves by the CTAB method [13]. A total of 465 SSR markers developed by USDA [14] and by the Kazusa DNA Research Institute [15] were used for screening of polymorphisms among the parents. SSR analysis was performed as previously described [9]. The markers were tested by Chi-square analyses for segregation in 1:1 ratio. A linkage map of the genotypic data for the 94 RILs was constructed using the MAPMAKER/EXP. ver. 3.0 [16] with the threshold LOD score of 3.0. Designation of linkage groups followed Cregan et al. [17].

### Molecular cloning

Total RNA was extracted from 200 mg of trifoliolate leaves of Harosoy and Nezumisaya using the TRIZOL Reagent (Invitrogen) according to the manufacturer’s instructions. cDNA was synthesized by reverse transcription of 5 μg of total RNA using the Superscript III First-Strand Synthesis System (Invitrogen) and an oligo(dT) primer according to the manufacturer’s instructions. The full-length cDNA was cloned by end-to-end PCR from Harosoy and Nezumisaya using a pair of PCR primers (Table 1) based on the genome sequence of US cultivar Williams 82 deposited in the soybean genome database (Phytozome, http://www.phytozome.net/soybean.php). The PCR mixture contained 0.5 μg of cDNA, 10 pmol of each primer, 5 pmol of nucleotides and 1 unit of ExTaq in 1 × ExTaq Buffer in a total volume of 25 μl. A 30 sec denaturation at 94°C was followed by 30 cycles of 30 sec denaturation at 94°C, 1 min annealing at 59°C and 1 min extension at 72°C. A final 7 min extension at 72°C completed the program. The 5′ upstream region of about 1.8 kb was amplified from Harosoy and Nezumisaya using GenomeWalker (Invitrogen) according to the manufacturer’s instructions. Primers used for nested PCR are shown in Table 1. The PCR products were cloned into the pCR 2.1 vector (Invitrogen) and sequenced.

**Table 1:** **PCR primers used in this study**

**Purpose**	**Target gene**	**Forward primer (5′-3′)**	**Reverse primer (5′-3′)**
Cloning of cDNA for sequencing	*GmF3G2″Gt*	AATCACGTCCTTTGCACATA	CAAAACCATTGCCATTCATG
Sequencing of cDNA	*GmF3G2″Gt*	AGGCTGTGCACTATTGTACT	TCACAATGCTCACAGCTTTA
		CCATATATGGATTACATTGG	CACTTCCAAAGCAACAATAA
		TTCTTGCACACCCTTCTGTA	GACAGAGCTAGCAGTACAAT
Cloning of 5′ upstream region^a^	*GmF3G2″Gt*		GTGTGTGTCTCTGGTGTAGTAATCATG
			CTCACACAAAACAGAGGTTCAATACGAGG
Sequencing of 5′ upstream region	*GmF3G2″Gt*	ACAACGGTCATATATGCATG	TGGCGGAGAGGAAGAAGATA
		ACAACGGTCATTTCTGCATG	CTCTCTGAAATGGATCCCAA
		GTCAACAATGAAGGACATGG	TAGAAATGCACGTAGTAGGT
			TCCACCACAATATGCTATGT
dCAPS analysis	*GmF3G2″Gt*	CCAAACTCACGAGGCTGGT	TCCTCCAAATCTAAAGTTGG
Cloning for functional analysis^b^	*GmF3G2″Gt*	GCCG*GAGCTC*ATGAAATCACGTCCTTTGC	GCCG*CTCGAG*TCAAATTCCCTCTACAATCTC
Real-time PCR	*GmF3G2″Gt*	AGAACCAAAGGATCTCCGTT	GTTTTTGCCAAGGAGATAAGT
	*actin*	GTCCTTTCAGGAGGTACAACC	CCACATCTGCTGGAAGGTGC

^a^Gene-specific primers for genome walking.

^b^Restriction sites incorporated in primers, *Sac*I site in forward primer and *Xho*I site in reverse primer are italicized.

### Sequencing analysis

Nucleotide sequences were determined with the BigDye terminator cycle method using an ABI3100 Genetic Analyzer (Applied Biosystems). Nucleotide sequences and the putative amino acid translations were analyzed with the BLAST program [18]. Intron/exon structure of the gene was estimated based on the comparison between the cDNA sequence and the corresponding genome sequences of Williams 82 deposited in the soybean genome database. Sequence alignment was performed with ClustalW (http://clustalw.ddbj.nig.ac.jp/index.php?lang=ja) using default settings. The amino acid sequences were aligned using ClustalW and the alignment was used to construct a phylogenetic tree using the neighbor-joining method with MEGA5 version 5.2.2 (http://www.megasoftware.net/) [19]. Bootstrap test of 1000 replications was performed. Using the structure of the grape flavonoid 3-*O-*glucosyltransferase (VvGT1, protein ID: 2C1Z) [20] as a template, homology modeling was conducted using GASH (Genetic-algorithm ASH) [21] program at the Protein Data Bank Japan (http://sysimm.ifrec.osaka-u.ac.jp/ash_service/). A model of GmF3G2"Gt-b was superimposed on 2C1Z using CueMol software (http://www.cuemol.org/en/).

### dCAPS analysis

A pair of PCR primers flanking a two-base substitution was designed (Table 1). A mismatched base G was incorporated in the forward primer to produce a *Kpn*I site in the amplified product of Harosoy. The base substitution in Nezumisaya eliminates the restriction site to generate a polymorphism. The PCR mixture contained 30 ng of genomic DNA, 5 pmol of each primer, 10 pmol of nucleotides and 1 unit of ExTaq in 1 x ExTaq Buffer in a total volume of 25 μl. After an initial 30 sec denaturation at 94°C, there were 30 cycles of 30 sec denaturation at 94°C, 1 min annealing at 56°C and 1 min extension at 72°C. A final 7 min extension at 72°C completed the program. The amplified products were digested with *Kpn*I, and the digests were separated on 8% nondenaturing polyacrylamide gels. After electrophoresis, the gel was stained with ethidium bromide and the DNA fragments were visualized under UV light.

### Heterologous expression and purification of recombinant GmF3G2″Gt protein

The entire coding region of *GmF3G2″Gt* was amplified from cDNA of Harosoy and Nezumisaya by PCR using the KOD -Plus- DNA polymerase (Toyobo) with high PCR fidelity and primers containing enzyme sites for *Sac*I and *Xho*I (Table 1). The PCR mixture contained 30 ng of genomic DNA, 10 pmol of each primer, 5 pmol of nucleotides, 2 mM of MgSO_4_ and 0.5 unit of KOD –Plus- in 1 × KOD –Plus- Buffer supplied by the manufacturer in a total volume of 25 μl. PCR conditions were identical to those in a previous report [9]. The PCR amplicon was digested with *Sac*I and *Xho*I and was cloned into the pCold ProS2 vector (Takara Bio). GmF3G2″Gt proteins were expressed and purified as described previously [22].

### Enzyme assay and MS/MS analysis

Enzyme assays, and MS and MS/MS analyses were conducted as previously described [9,23]. The ESI source was operated in negative ionization mode.

### Gene expression assays

cDNA was synthesized by reverse-transcription of 5 μg of total RNA from the parents at R6 stage in three replications using the Superscript III First-Strand Synthesis System and an oligo d(T) primer. Primer sequences are exhibited in Table 1. PCR conditions were identical to those in a previous report [9]. Expression levels of the soybean *actin* gene (GenBank accession number: J01298) [24] were used to normalize target gene expression and compared by *t*-test using Statistica 03J (StatSoft). PCR products were cloned into the pCR 2.1 vector. The nucleotide sequence of eight clones from each of the cultivars was determined.

## Results

### Flavonol glycoside composition

**Figure 1:**
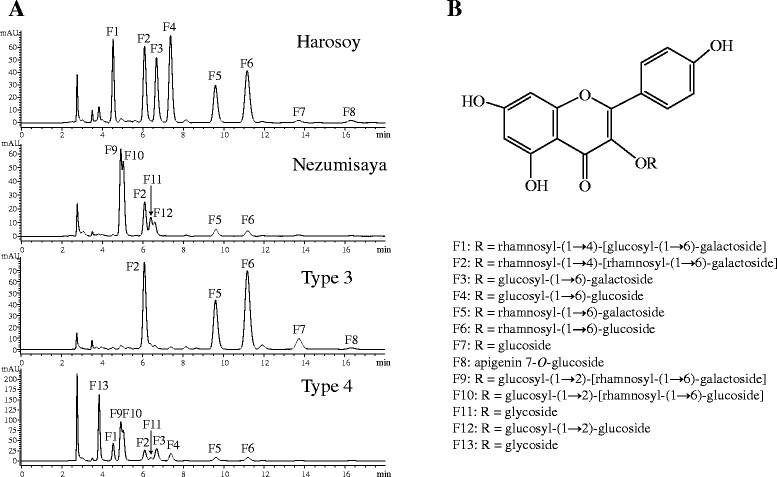
HPLC chromatogram of MeOH extracts from leaves of soybean cultivars Nezumisaya, Harosoy and recombinant inbred lines derived from a cross of the cultivars, and chemical structure of flavonol glycosides corresponding to HPLC peaks. **(A)** HPLC chromatogram. 100 mg of trifoliolate leaves was extracted with 1 ml of MeOH. Eluent: phosphoric acid/acetonitrile/H_2_O (0.2:18:82). Flow-rate: 1.0 ml/min. Injection: 10 μl. Detection: 350 nm. **(B)** Chemical structure.

HPLC chromatograms of Harosoy and Nezumisaya are shown in Figure 1A. Harosoy had eight primary peaks corresponding to FGs, viz., 4.5 (F1), 6.1 (F2), 6.7 (F3), 7.4 (F4), 9.6 (F5), 11.2 (F6), 13.7 (F7) and 16.3 min (F8). Nezumisaya had seven primary peaks, viz., 4.9 (F9), 5.0 (F10), 6.1 (F2), 6.4 (F11), 6.6 (F12), 9.6 (F5) and 11.2 (F6). Based on comparison with authentic specimens, the peaks correspond to the followings FGs: F1, kaempferol 3-*O*-rhamnosyl-(1 **→** 4)-[glucosyl-(1 **→** 6)-galactoside]; F2, kaempferol 3-*O-*rhamnosyl-(1 **→** 4)-[rhamnosyl-(1 **→** 6)-galactoside]; F3, kaempferol 3-*O*-glucosyl-(1 → 6)-galactoside; F4: kaempferol 3-*O*-glucosyl-(1 → 6)-glucoside; F5, kaempferol 3-*O*-rhamnosyl-(1 **→** 6)-galactoside; F6, kaempferol 3-*O*-rhamnosyl-(1 **→** 6)-glucoside; F7, kaempferol 3-*O*-glucoside; F8, apigenin 7-*O*-glucoside; F9, kaempferol 3-*O*-glucosyl-(1 **→** 2)-[rhamnosyl-(1 **→** 6)-galactoside]; F10, kaempferol 3-*O*-glucosyl-(1 **→** 2)-[rhamnosyl-(1 **→** 6)-glucoside]; F11, kaempferol glycoside; F12, kaempferol 3-*O*-glucosyl-(1 **→** 2)-glucoside; F13, kaempferol glycoside (Figure 1B).

The peaks F9, F10 and F12 that were specific to Nezumisaya corresponded to FGs having glucose at the 2″-position of glucose or galactose that is bound to the 3-position of kaempferol, whereas FGs with 2″-glucose were absent in Harosoy. The peaks F1, F3 and F4 that were specific to Harosoy corresponded to FGs having glucose at the 6″-position of glucose or galactose that is bound to the 3-position of kaempferol, whereas FGs with 6″-glucose were absent in Nezumisaya.

### Inheritance of flavonol glycoside composition

Among the 94 RILs cultivated in field, 3 RILs matured too early for sampling. The remaining 91 RILs were subjected to HPLC analysis. Among the RILs, 22 RILs had peaks of the Harosoy-type, and 23 RILs had peaks of the Nezumisaya-type. 18 RILs had a peak distribution designated as ‘type 3’; this lacked three peaks (F1, F3 and F4) compared with the Harosoy-type (Figure 1A). Further, 28 RILs were designated as ‘type 4’; these had a mixture of peaks from both cultivars in addition to a unique peak, F13. The segregation fitted to a 1:1:1:1 ratio, suggesting that two genes control the FG pattern in the RIL (χ^2^ = 2.23, P = 0.53).

One of the genes may be responsible for attachment of glucose to the 2″-position and it probably encodes a flavonol 3-*O*-glucoside/galactoside (1 → 2) glucosyltransferase. Nezumisaya may have a dominant while Harosoy may have a recessive allele of the gene. The other gene may be involved in the attachment of glucose to the 6″-position and probably encodes a flavonol 3-*O*-glucoside/galactoside (1 → 6) glucosyltransferase. Harosoy may have a dominant while Nezumisaya may have a recessive allele of the gene. RILs of ‘type 3’, in which FGs with 2″-glucose and FGs with 6″-glucose were absent, may have double-recessive alleles of the two genes. RILs of ‘type 4’, in which FGs with 2″-glucose and FGs with 6″-glucose were present, may have double-dominant alleles.

### Linkage mapping of flavonol glycoside gene

**Figure 2:**
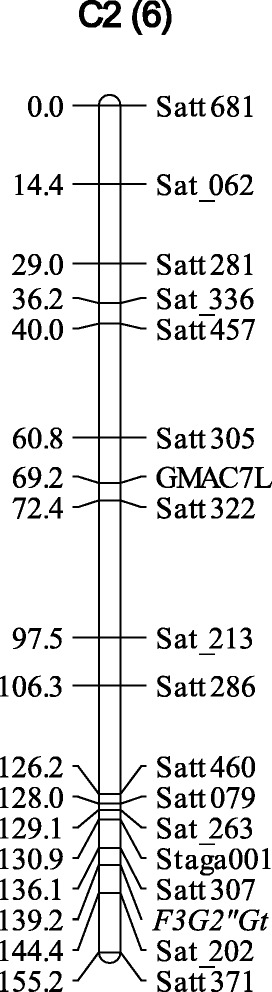
Linkage mapping of *F3G2″Gt* using recombinant inbred lines derived from a cross between soybean cultivars Nezumisaya and Harosoy. The name of the linkage group is indicated at the top followed by the chromosome number in parenthesis. Distances (cM) of markers from the top of the linkage group are shown on the left.

Among the 465 SSR markers tested, 185 markers that exhibited polymorphism between the parents and distinctly segregated in the RILs were used for linkage mapping. A total of 99 markers were classified into 28 linkage groups spanning 2,172 cM. For mapping of the gene responsible for attachment of glucose to the 2″-position, RILs having FG composition of the Nezumisaya type and ‘type 4’ were considered to have the genotype of Nezumisaya, whereas RILs having FG composition of the Harosoy type and ‘type 3’ were considered to have the genotype of Harosoy. Linkage mapping revealed that the gene responsible for attachment of glucose to the 2″-position was located in the molecular linkage group C2 (chromosome 6) between Satt307 and Sat_202 (Figure 2).

### Molecular cloning of flavonol glycoside gene

**Figure 3:**
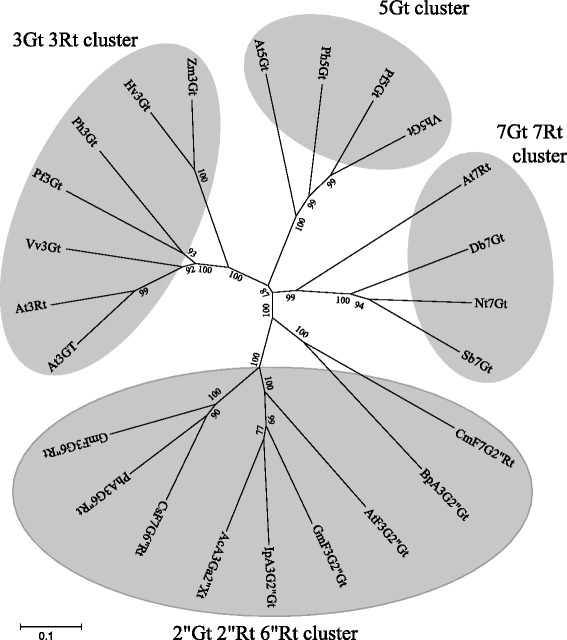
Unrooted molecular phylogenetic tree of some flavonoid glycosyltransferases. Bar represents 0.1 amino acid substitutions/site. The GenBank accession numbers for the sequences are shown in parentheses: At3Rt (NM_102790); At3Gt (NM_121711); Vv3Gt (AF000371); Ph3Gt (AB027454); Pf3Gt (AB002818); Hv3Gt (X15694); Zm3Gt (X13501); At5Gt (NM_117485); Pf5Gt (AB013596); Vh5Gt (BAA36423); Ph5Gt (AB027455); Db7Gt (CAB56231); Nt7Gt (AAB36653); Sb7Gt (BAA83484); At7Rt (AY093133); CmF7G2*″*Rt (AAL06646); CsF7G6″Rt (ABA18631); IpA3G2″Gt (AB192315); PhA3G6″Rt (X71059); BpA3G2″Glt (AB190262); AcA3Ga2″Xt (FG404013); AtF3G2″Gt (Q9FN26). Gt, glucosyltransferase; Rt, rhamnosyltransferase; Xt, xylosyltransferase; Glt, glucuronosyltransferase. Ac, *Actinidia chinensis*; At, *Arabidopsis thaliana*; Bp, *Bellis perennis*; Cm, *Citrus maxima*; Cs, *Citrus sinensis*; Db, *Dorotheanthus bellidiformis*; Gm, *Glycine max;* Hv, *Hordeum vulgare*; Ip, *Ipomoea purpurea*; Nt, *Nicotiana tabacum*; Pf, *Perilla frutescens*; Ph, *Petunia hybrida*; Sb, *Scutellaria baicalensis*; Vh, *Verbena hybrida*; Vv, *Vitis vinifera*; Zm, *Zea mays*.

**Figure 4:**
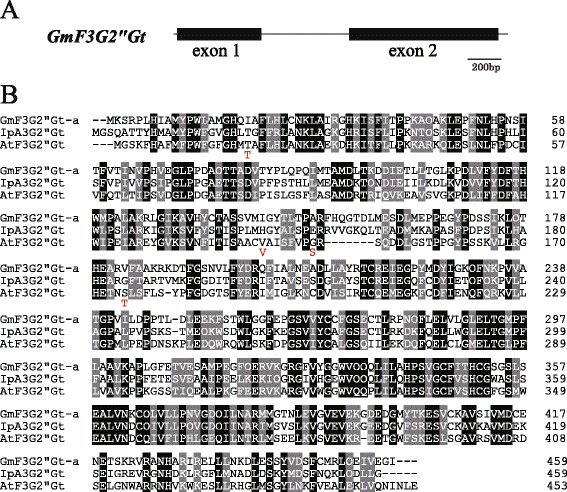
Gene structure and amino acid alignment of *GmF3G2″Gt*. **(A)** Intron-exon structure of *GmF3G2″Gt* gene. **(B)** Amino acid alignment of soybean *GmF3G2″Gt-a*, morning glory *Ip3GGT* encoding anthocyanin 3-*O*-glucoside (1 → 2) glucosyltransferase and Arabidopsis UGT79B6 that functions as a flavonol 3-*O*-glucoside (1 → 2) glucosyltransferase. Identical amino acids are in white font highlighted in black, similar amino acids are in white font highlighted in gray. Four amino acids that differed in *GmF3G2″Gt-b* are exhibited below the aligned sequence in red font.

Survey of the genome sequence of a US cultivar Williams 82 suggested the existence of a gene similar to the GT gene, Glyma06g43880 between Satt307 and Sat_202. The entire coding region of Glyma06g43880 was amplified by PCR and cloned. Sequence analysis revealed that the open reading frame of Glyma06g43880 is 1380 bp long encoding 459 amino acids. We designated the gene as *GmF3G2″Gt. GmF3G2"Gt* belongs to the family 1 glycosyltransferase, and it was designated as UGT79B30 by the UGT Nomenclature Committee [25]. The flavonoid glycosyltransferase phylogenetic tree suggested that *GmF3G2″Gt* belongs to the flavonoid glycoside glycosyltransferase (GGT) gene cluster (Figure 3). BLAST analysis suggested that it had a 55% amino acid similarity with *Ip3GGT* of morning glory encoding anthocyanin 3-*O*-glucoside (1 → 2) glucosyltransferase [26] and 45% similarity with Arabidopsis UGT79B6 that was recently identified to function as a flavonol 3-*O*-glucoside (1 → 2) glucosyltransferase [22] (Figure 3). Comparison with the genome sequence of Williams 82 suggested that *GmF3G2″Gt* had one intron (Figure 4A). Eight nucleotides were polymorphic between Harosoy and Nezumisaya; consisting of six single nucleotide polymorphisms (SNPs) and one two-nucleotide substitution. The nucleotide polymorphism resulted in four amino acid substitutions between the cultivars (amino acid positions, 20, 142, 149 and 183) (Figure 4B). The cDNA fragments generated from Nezumisaya and Harosoy were designated as *GmF3G2″Gt-a* and *GmF3G2″Gt-b*, respectively. The 5′ upstream region of about 1.8 kb was amplified by PCR from Harosoy, whereas the corresponding region could not be amplified from Nezumisaya. So we cloned the 5′ upstream region of both cultivars by genome walking. The nucleotide sequences of the 5′ upstream region, exons and introns of Harosoy were identical with those of Williams 82. In contrast, Nezumisaya had many indels and substitutions in the 5′ upstream region compared with Harosoy (Additional file 1: Figure S1).

### dCAPS analysis

**Figure 5:**
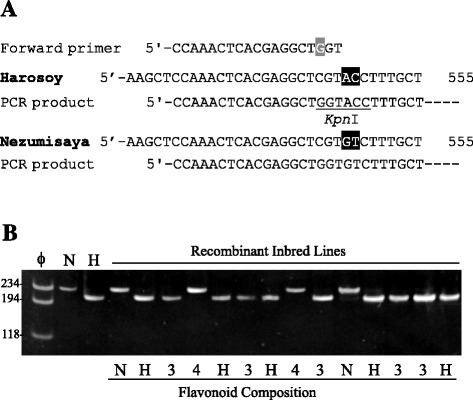
Outline and results of dCAPS analysis of *GmF3G2″Gt* in soybean. **(A)** Schematic presentation of dCAPS analysis. Partial nucleotide sequences around the region polymorphic between Harosoy and Nezumisaya are exhibited. A mismatched base in the forward primer is highlighted in gray. Polymorphic nucleotides are highlighted in black. **(B)** Results of dCAPS analysis of the parents and the recombinant inbred lines derived from a cross between Nezumisaya and Harosoy. PCR products amplified with dCAPS primers were digested by *Kpn*I and the digests were separated on an 8% polyacrylamide gel. ϕ, molecular marker ϕx174/*Hae*III; N, Nezumisaya; H, Harosoy. FG pattern of the recombinant inbred lines is exhibited below the gel. H, Harosoy-type; N, Nezumisaya-type, 3, type 3; 4, type 4. The migration of size markers (bp) is shown to the left of the gel.

PCR products with molecular size of 230 bp were amplified with the dCAPS primers in Nezumisaya and Harosoy (Figure 5). *Kpn*I digestion generated a band of 213 bp in Harosoy, whereas the PCR amplicon of Nezumisaya was unaffected. Banding patterns co-segregated with FG patterns; RILs with FGs of the Harosoy type and ‘type 3’ had bands of Harosoy type, whereas RILs with FGs of the Nezumisaya type and ‘type 4’ had bands of Nezumisaya type (Figure 5).

### *In vitro characterization of recombinant* GmF3G2″Gt

**Figure 6:**
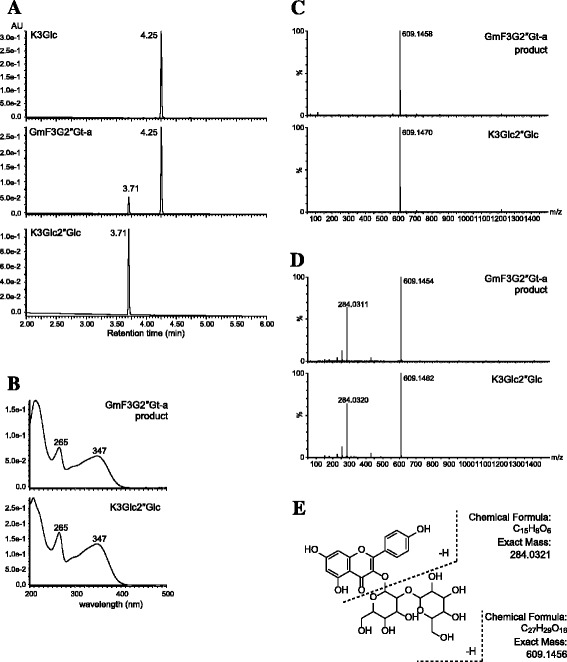
Identification of reaction product of GmF3G2″Gt-a (cultivar Nezumisaya). **(A)** Elution profiles of the standards (kaempferol 3-*O*-glucoside and kaempferol 3-*O*-sophoroside) and reaction product of GmF3G2″Gt-a protein. **(B)** UV spectra of the standard (kaempferol 3-*O*-sophoroside) and reaction product of GmF3G2″Gt-a protein. Mass spectra **(C)** and MS/MS spectra **(D)** of the standard (kaempferol 3-*O*-sophoroside) and reaction product of GmF3G2″Gt-a protein. **E**, The MS/MS fragmentation for kaempferol 3-*O*-sophoroside. K3Glc, kaempferol 3-*O*-glucoside; K3Glc2″Glc, kaempferol 3-*O*-sophoroside.

The GmF3G2″Gt recombinant protein of Nezumisaya (GmF3G2″Gt-a) and Harosoy (GmF3G2″Gt-b) were expressed in *E. coli* as a His/ProS2 fusion and purified. The GmF3G2″Gt proteins were used for enzymatic assays after cleavage of the His/ProS2 tag. GmF3G2″G-a and GmF3G2″G-b converted kaempferol 3-*O*-glucoside to kaempferol 3-*O*-sophoroside as confirmed by comparison of retention time, UV spectra and MS/MS ionization with the standard compound (Figure 6, Additional file 2: Figure S2). GmF3G2″Gt-a showed a broad activity for kaempferol/quercetin 3-*O*-glucoside/galactoside derivatives (Table 2). However, GmF3G2″Gt-a did not glucosylate kaempferol 3-*O*-rhamnosyl-(1 → 4)-[rhamnosyl-(1 → 6)-glucoside] and 3-*O*-rhamnosyl-(1 → 4)-[glucosyl-(1 → 6)-glucoside]. GmF3G2″Gt-a had a higher preference for UDP-glucose than UDP-galactose, with only 3% activity relative to that for UDP-galactose. No UGT activity was detected for UDP-arabinose and UDP-glucuronic acid. GmF3G2″Gt-b also showed similar substrate specificity (Additional file 3: Table S1). Accordingly, GmF3G2″Gt-a and GmF3G2″Gt-b were defined as flavonol 3-*O*-glucoside/galactoside (1 → 2) glucosyltransferases.

**Table 2:** **Substrate specificity of** ***GmF3G2″Gt*** **in cultivar Nezumisaya**

	**Relative activity (%)**
*Sugar acceptor* ^a^	
Kaempferol (Kae) 3-*O*-glucoside^b^	100.0 ± 5.3
Kae 3-*O*-galactoside	73.7 ± 0.5
Kae 3-*O*-rhamnosyl-(1 → 6)-glucoside	42.4 ± 3.7
Kae 3-*O*-rhamnosyl-(1 → 4)-[rhamnosyl-(1 → 6)-glucoside]	N.D.
Kae 3-*O*-rhamnosyl-(1 → 4)-[glucosyl-(1 → 6)-glucoside]	N.D.
Kae 3-*O*-glucoside-7-*O*-rhamnoside	23.8 ± 1.1
Quercetin (Que) 3-*O*-glucoside	75.2 ± 4.9
Que 3-*O*-galactoside	78.9 ± 2.4
Que 3-*O*-rhamnosyl-(1 → 6)-glucoside	25.4 ± 1.3
*Sugar donor* ^c^	
UDP-glucose	100.0 ± 5.2
UDP-galactose	3.3 ± 0.1
UDP-arabinose	N.D.
UDP-glucuronic acid	N.D.

N.D., not detected.

^a^The reactions were performed with UDP-glucose as the sugar donor.

^b^The product was identified based on comparison with the pertinent standard.

^c^The reactions were performed with kaempferol 3-*O*-glucoside.

### Gene expression

The accumulation of *GmF3G2″Gt* transcript in leaves of Nezumisaya and Harosoy was analyzed by real-time PCR. Survey of soybean genome sequence revealed the existence of a gene similar to *GmF3G2″Gt*, Glyma12g14050 with nucleotide similarity of 93% in chromosome 12 (molecular linkage group H). It was difficult to design primers for *GmF3G2″Gt* based on the nucleotide sequence of its coding region. So, we designed primers for real-time PCR based on distinct sequences from the 5' untranslated region. At the R6 stage, the transcript level of *GmF3G2″Gt* in leaves of Harosoy was 14.6% of that in Nezumisaya (*t* = 3.60*). The PCR products from Nezumisaya and Harosoy were ascertained to be derived from the expected genome region by sequencing.

## Discussion

Harosoy and Nezumisaya are soybean cultivars that have gray pubescence and they both have significant deposits of kaempferol derivatives in their leaves. However, these cultivars differ in their FG composition. HPLC comparison with authentic samples suggested that FGs having glucose at the 2″-position of glucose or galactose that is bound to the 3-position of kaempferol were present in Nezumisaya, whereas FGs of Harosoy were devoid of 2″-glucose. Conversely, FGs having glucose at the 6″-position of glucose or galactose that is bound to the 3-position of kaempferol were present in Harosoy, whereas these FGs were absent in Nezumisaya. Apigenin 7-*O*-glucoside almost exclusively deposits in the cytoplasm of gray pubescence [27], suggesting that the peak F8 may have been derived from pubescence on leaves.

Genetic analysis suggested that two genes control the FG pattern. One of the genes may be responsible for attachment of glucose to the 2″-position, and it probably encodes a flavonol 3-*O*-glucoside/galactoside (1 → 2) glucosyltransferase. Nezumisaya may have a dominant, whereas Harosoy may have a recessive allele of the gene. The gene was mapped in molecular linkage group C2 (chromosome 6) between Satt307 and Sat_202. Judging from the relative location with SSR markers, its position was similar to that of *Fg3* reported by Buzzell [28]. The other gene may be involved in the attachment of glucose to the 6″-position, and it probably encodes a flavonol 3-*O*-glucoside/galactoside (1 → 6) glucosyltransferase. The gene may correspond to the *Fg1* gene reported previously [2]. Harosoy may have a dominant whereas Nezumisaya may have a recessive allele of the gene. The ‘type 3’ may have double-recessive alleles whereas the ‘type 4’ may have double-dominant alleles for the two FG genes. F13 probably corresponds to a kaempferol glycoside having both 2″-glucose and 6″-glucose.

A survey of the genome sequence of US cultivar Williams 82 suggested that a putative GT gene, Glyma06g43880 existed between Satt307 and Sat_202. We cloned the cDNA and designated it as *GmF3G2″Gt*. The open reading frame of *GmF3G2″Gt* is 1380 bp long encoding 459 amino acids. *GmF3G2″Gt* belongs to the GGT gene cluster. *GmF3G2″Gt* of Nezumisaya had an amino acid similarity of 55% with the *Ip3GGT* of morning glory encoding a 3-*O*-glucoside (1 → 2) glucosyltransferase that also attaches glucose to the 2″-position of glucose that is bound to the 3-position of anthocyanidins [26]. It also has 45% similarity with Arabidopsis UGT79B6 that functions as a flavonol 3-*O*-glucoside (1 → 2) glucosyltransferase [22]. The genome sequence of Williams 82 suggests the existence of a gene (Glyma12g14050) having 93% of nucleotide identity with Glyma06g43880 in chromosome 12. However, the gene responsible for the attachment of glucose to the 2″-position was not mapped to chromosome 12 in the current as well as in a previous study [28]. Thus, based on our experimental data, Glyma12g14050 may not be responsible for the attachment of glucose to the 2″-position.

In the coding region of *GmF3G2″Gt*, six SNPs and one two-base substitution existed between Harosoy and Nezumisaya, resulting in four amino acid substitutions. The dCAPS marker that was used to discriminate the two-base substitution co-segregated with the FG patterns, suggesting that *GmF3G2″Gt* might correspond to a flavonol 3-*O*-glucoside/galactoside (1 → 2) glucosyltransferase (*Fg3*) gene.

The recombinant *GmF3G2″Gt-a* protein converted UDP-glucose and kaempferol 3-*O*-glucoside to kaempferol 3-*O*-sophoroside, proving that *GmF3G2″Gt* encodes a flavonol 3-*O*-glucoside/galactoside (1 → 2) glucosyltransferase. The protein did not discriminate between glucose and galactose attached to the 3-position of kaempferol based on the results of HPLC analysis. Hence, it is similar to the soybean flavonol 3-*O*-glucoside/galactoside (1 → 6) rhamnosyltransferase [9]. GmF3G2″Gt-a protein showed a broad activity for kaempferol/quercetin 3-*O*-glucoside/galactoside derivatives, but it did not glucosylate kaempferol 3-*O*-rhamnosyl-(1 → 4)-[rhamnosyl-(1 → 6)-glucoside] and 3-*O*-rhamnosyl-(1 → 4)-[glucosyl-(1 → 6)-glucoside]. This suggests that 2″-glucosylation of flavonol 3-*O*-glycoside is irreconcilable with 4″-glycosylation. This hypothesis is supported by the flavonoid profiles of Nezumisaya and ‘type 4’ in which FGs with 2″ and 4″-double glycosylation are lacking. Further, leaves of seven soybean accessions contained FGs with double glycosylation of 2″ and 6″, or 4″ and 6″, but lacked FGs with double glycosylation of 2″ and 4″ [11].

Though four amino acids were substituted, the recombinant *GmF3G2″Gt-b* protein also had a flavonol 3-*O*-glucoside/galactoside (1 → 2) glucosyltransferase activity. A structural modeling of GmF3G2″Gt using the 3D structure of a grape flavonoid 3-*O*-glucosyltransferase, VvGT1, as a template indicated that three substituted amino acids (V142, S149, T183 in GmF3G2″Gt-b) were located far from the enzyme active sites. The other amino acid (T20) was relatively close to the uracil ring of UDP-2-deoxy-2-fluoro-glucose (6.55 Å) but far from the active center. The threonine residues corresponding to T20 in GmF3G2″Gt-b were also conserved in flavonoid 3-*O*-glucoside: (1-2) glucosyltransferase, such as AtF3G2″Gt and IpA3G2″Gt. These data suggest that four substituted amino acid residues in GmF3G2″Gt-b are unlikely to affect enzyme activity. The transcript level of *GmF3G2″Gt* in leaves of Harosoy was about 15% of that in Nezumisaya. GmF3G2″Gt protein possibly have a threshold for catalysis and that of Harosoy is lower than the threshold. To confirm the hypothesis, further studies such as association analysis between gene expression and FG amounts among tissues or developmental stages may be necessary. Indels and substitutions in the 5′ upstream region may be responsible for the differences in expression level. Promoter assays may be necessary to determine which polymorphism is critical for gene expression.

The nucleotide sequences in the 5′ upstream region, exons and intron of *GmF3G2″Gt* from Harosoy were identical to those of Williams 82. The HPLC chromatogram of leaf methanol extracts of Williams 82 was identical to that of US cultivar Clark which has an allelic combination of *fg1Fg2fg3Fg4* [6], suggesting that Williams 82, Clark and Harosoy might have a recessive allele at the *Fg3* locus (Di et al., unpublished results, 2014). The results are consistent with our hypothesis that Nezumisaya has a dominant and Harosoy has a recessive allele for the *Fg3* gene. Based on the HPLC profiles, Harosoy may have an allelic combination of *Fg1Fg2fg3Fg4* while Nezumisaya may have a combination of *fg1Fg2Fg3Fg4*. This is consistent with a previous report that Harosoy has an allelic combination of either *Fg1Fg2fg3* or *fg1Fg2fg3* depending on the line [29].

Multiple alignment of GGTs (three G6″GTs and six G2″GTs) suggested 32 amino acids specific to G6″GTs and 4 amino acids specific to G2″GTs (Additional file 4: Figure S3). There are characteristic amino acids in position 36 (V in G6″GTs and I in G2″GTs) and amino acid position 347 (Y in G6″GTs and F in G2″GTs) in the plant secondary product glycosyltransferase motif around the C-terminal region [30]. Site-directed mutagenesis may reveal which amino acids are responsible for glycosylation of specific positions of the sugar moiety in flavonoids. Amino acids responsible for sugar donor specificity (Ser-138 or Gly-138) were identified in a GGT gene involved in saponin biosynthesis [31]. Multiple alignment indicated that all three flavonoid G2″GTs had Thr at this position, suggesting a possibility that the amino acid is also involved in sugar donor specificity in flavonoid GGTs (Additional file 4: Figure S3). The *GmF3G2″Gt* had only one intron, consistent with FG genes of other plant species, where either existence of no or one intron is predominant [32].

Cloning of *Fg1* encoding flavonol 3-*O*-glucoside/galactoside (1 → 6) glucosyltransferase and *Fg4* encoding flavonol 3-*O*-glucoside/galactoside (1 → 2) rhamnosyltransferase may help clarify the recognition mechanisms for the hydroxyl group of the sugar moiety (at 2″- or 6″-position), ability to specify the nature of the sugar (glucose or rhamnose), and molecular evolution of UGT genes. In addition, another gene having the function of flavonol 3-*O*-galactoside (1 → 4) rhamnosyltransferase remains to be cloned. Cloning of the entire gene set may be necessary to understand FG biosynthesis in soybean. Development of near-isogenic lines by incorporating various combinations of alleles for *Fg* genes into common genetic backgrounds may be necessary to obtain information on the dosage effect of *Fg* genes in relation to morphology, productivity and other agronomic characters in soybean.

## Conclusions

FGs having glucose at the 2″-position of glucose or galactose that is bound to the 3-position of kaempferol were present in Nezumisaya, whereas FGs of Harosoy were devoid of 2″-glucose. Conversely, FGs having glucose at the 6″-position of glucose or galactose that is bound to the 3-position of kaempferol were present in Harosoy, whereas these FGs were absent in Nezumisaya. Two genes control the FG pattern; the first is responsible for the attachment of glucose to the 2″-position, and encodes a flavonol 3-*O*-glucoside/galactoside (1 → 2) glucosyltransferase. Nezumisaya has a dominant whereas Harosoy has a recessive allele of the gene. A candidate gene *GmF3G2″Gt* was located in the molecular linkage group C2 (chromosome 6). The open reading frame of *GmF3G2″Gt* is 1380 bp long encoding 459 amino acids with four amino acid substitutions among the cultivars. The *GmF3G2″Gt* recombinant protein converted kaempferol 3-*O*-glucoside to kaempferol 3-*O*-sophoroside. Hence, *GmF3G2″Gt* encodes a flavonol 3-*O*-glucoside/galactoside (1 → 2) glucosyltransferase and corresponds to the *Fg3* gene. Based on substrate specificity, 2″-glucosylation of flavonol 3-*O*-glycoside may be irreconcilable with 4″-glycosylation in soybean leaves.

### Availability of supporting data

Sequence data from this article have been deposited at the DDBJ Data Libraries under accession numbers LC017844 (cDNA of *GmF3G2″Gt-a*), LC017845 (cDNA of *GmF3G2″Gt-b*), LC017916 (5′ upstream region of *GmF3G2″Gt-a*) and LC017917 (5′ upstream region of *GmF3G2″Gt-b*).

## Additional files

Additional file 1: Figure S1. Alignment of the 5′ upstream region of *GmF3G2″Gt* gene in soybean cultivars Harosoy and Nezumisaya. Polymorphic nucleotides are shown in red font. Coding sequence is underlined. Additional file 2: Figure S2. Identification of reaction product of GmF3G2″Gt-b (cultivar Harosoy). (A) Elution profiles of the standards (kaempferol 3-*O*-glucoside and kaempferol 3-*O*-sophoroside) and reaction product of GmF3G2″Gt-b protein. (B) UV spectra of the standard (kaempferol 3-*O*-sophoroside) and reaction product of GmF3G2″Gt-b protein. Mass spectra (C) and MS/MS spectra (D) of the standard (kaempferol 3-*O*-sophoroside) and reaction product of GmF3G2″Gt-b protein. K3Glc, kaempferol 3-*O*-glucoside; K3Glc2″Glc, kaempferol 3-*O*-sophoroside. Additional file 3: Table S1. Substrate specificity of *GmF3G2″Gt* in cultivar Harosoy. Additional file 4: Figure S3. Multiple alignment of flavonoid glycoside glycosyltransferases (GGTs). The plant secondary product glycosyltransferase motif is underlined. Amino acid residues conserved in flavonoid GGTs are in white font highlighted in black. Amino acid residues conserved in flavonoid G6″GTs and G2″GTs are in white font highlighted in red and blue, respectively. Position of amino acid responsible for sugar specificity in *Sg-1* glycosyltransferase is shown by white triangle. Thr residues conserved in G2″GTs are in white font highlighted in green.

## Abbreviations

FG: Flavonol glycoside
GGT: Flavonoid glycoside glycosyltransferase
GT: Glycosyltransferase
HPLC: High performance liquid chromatography
RIL: Recombinant inbred line
SNP: Single nucleotide polymorphism
UGT: UDP glycosyltransferase
