# Supplementary material for: Linkage mapping, molecular cloning and functional analysis of soybean gene Fg3 encoding flavonol 3-O-glucoside/galactoside (1 → 2) glucosyltransferase
Source: BMC Plant Biol. 2015 May 23;15:126. doi: 10.1186/s12870-015-0504-7 (PMC4494776; doi:10.1186/s12870-015-0504-7)
Supplement: Additional file 1: Figure S1. — Alignment of the 5′ upstream region of GmF3G2″Gt gene in soybean cultivars Harosoy and Nezumisaya. Polymorphic nucleotides are shown in red font. Coding sequence is underlined. [file 12870_2015_504_MOESM1_ESM.pdf]

```
Nezumisaya    AATTTGTGGGTTTTGAGAACTACCATTGGTAGGTGGCTCTACATTGGATGAAGGATGCAT -1782
Harosoy       AATTTGTGGGTTTTGAGAACTACCGTTGGTAGGTGGCTCTACATTGGATGAAGGATGCAT -1785

Nezumisaya    ATATCACATGTGTGGGGGTGGTGGATACATAATATATTGTGGTGGATTTTGAGAATTTTC -1722
Harosoy       ATACCACATGTGTGGGGGTGGTGGATACATAATATATTGTGGTGGATTTTGAGAATTTTC -1725

Nezumisaya    ACATAGCATATTGTGGTGGATTTTGAAAATTTTCACTGGTATGGGGTGGTCGATACATAA -1662
Harosoy       ACATAGCATATTGTGGTGGATTTTAAAAATTTTCACTGGTATGGGGTGGTCGATACATAA -1665

Nezumisaya    CATATTGCGGTGGATTTTGAAAATTTTCACTAGTAGGAAGTGATTGATTTTGCAAATAAC -1602
Harosoy       CATATTGCGGTGGATTTTGAGAATTTTCA-TAGTAGGGAGTGATTGATTTTGCAAATAAC -1606

Nezumisaya    TATAGTAACATTGATAATTATACTGATTGGGATCCATTTCAGAGAGAATAAAAAAATTTG -1542
Harosoy       TATAGTAACATTGATAATTATACTGATTGGGATCCATTTCAGAGAGAATAAAAAAAATTG -1546

Nezumisaya    ACACTTGATAGCAACAACAACAAAAAAAAATTAGCAGTACAAGAACTATGCAAAAAATGTT -1482
Harosoy       ACACTTGATAGCAACAACAACAAAAAAAAATTAGCAGTACAAGAACTATGCAAAACATGTT -1486

Nezumisaya    CTACCGTGCACACATTTATAGTGAATAGTGTTGTACAGTGAAAAAATACTACACAACGGT -1422
Harosoy       GTACCGTGCACAAATTTATAATGAATAGTGTAGTACAGTGAAAAAATACTACACAACGGT -1426

Nezumisaya    CATATATGCATGTTTAACGGTATTATTTTTCATATCAACGGTACATAACAACGATAACAT -1362
Harosoy       CATTTCTGCATGTTCAACGGTATTA-TTTTCAGATGAACGGTACATAACAACGGTAACAT -1367

Nezumisaya    TTCCATCAACGTTCAAAATCCTTTCCTCATTAATTACCTAGGCTGTTCAACAGTCCATAT -1302
Harosoy       TTCCATCAACGTTCAAAATCC-TTCCTCATTAATTACCTAAGTTGTTCAATAGTCCATAT -1308

Nezumisaya    TCATCCTCATTAATAAAATGGATGGTCCAGATCGTGTCACCTCATTAATTACCTAAAGCT -1242
Harosoy       TCATCCTCATTAATCAAATGGATGGTCCAGATCGTGTCACCTTATTAATTACCTAAAGCT -1248

Nezumisaya    ACCTACTACATGCATTTCTATCTTCTCGAGAGAACATTTTCTCTCTTCACCACCTCCAGA -1182
Harosoy       ACCTACTACGTGCATTTCTATCTTCTCGAGAGAACATTTTCTCTCTTCACCACCTCCAGA -1188

Nezumisaya    AGCTTCCCCCTCTGCTCCTATCTATCTTCTTCCTCTCCGCCATGGCCCAATCATCTCGTG -1122
Harosoy       AGCTTCCCCCTCTGCTCCTATATATCTTCTTCCTCTTCGCCATGGCCCATTCGTCTCGTG -1128

Nezumisaya    TGACCTTTGTCGGCATCAACACCTCCGACCACCACCGCAACACCTTCGTGTCACCACACC -1062
Harosoy       TGACCTTTGTCGGCATCAACACCTCCGGCCACCACCGCAACACCTTCGTGTCACCACACC -1068

Nezumisaya    TTCGTCTGGTCTCACCTTCACTTCGCGTCACCACCTCTGGCCATGGTAGTGTCTCGCGTC -1002
Harosoy       TTTGTCTGGCCTTACCTTCACTTCGCGTCACCACCTCTGGCCATGGTAGTGTCTCGCATC -1008

Nezumisaya    ACCTTTGTTGGCGTCAACAATGAAGGACATGGTCGCGTCTCCAGTCATGGTCTTGTCTCC  -942
Harosoy       ACCTTTGCTGGGGTCAACAATGAAGGACATGGTCGCGTCTCCGGTCATGGTTTCATCTTC  -948

Nezumisaya    GGCATGGTGCGTTTGTTGTCAGAATTGTTGTGTTTGATTTTCTCCTTTTTCTCCTTTACT  -882
Harosoy       GGCATGGTGCGTTTGTCGTCACAATTGTTGCGTTTGATTTTTTCCTTTTTCTCCTTTACT  -888

Nezumisaya    GTTTTGCTTGAATCTAGATTTAAATCACATTTTTTTTTCAAATTTGATGATTTTTGGGAT  -822
Harosoy       GTTTTGCTTGAATCTAGATTTAAATCACTTTTTTTTTTTAAATTTGATGATTTTTGGGAT  -828

Nezumisaya    TTATTGTGATTGTTCAATGAGTCTATACGTTCCCTATTCTTTCCTTGGACAATTATTTTT  -762
Harosoy       TTATTGTGGTTGTTCAACGAGTCTATACGTTTTGTGTTCTTTCCTTGGATAGTTATTTTT  -768

Nezumisaya    CCTCGTATTGAACCTCTGTTTTGTGTGAGAAAATCAATTTTTTAATGGTTAAATATTTGT  -702
Harosoy       CCTCGTATTGAACCTCTGTTTTGTGTGAGAAAATCAATTTTTTAATGATTAGATATTTGT  -708

Nezumisaya    TATGTATGTGAAAATCATTTTTAAAATAGGTTCTCTTTCCCTAAGAACATCAAAGTTTAT  -642
Harosoy       TATGTATGTGAAAATCATTTTTAAAATAGGTTCTCTTTCCGTAAGAACATCAAAGTTTAT  -648

Nezumisaya    GATTATGTTTTCCCTTTAATTTTTGTGGCCGTTATGGTTTTAACTTTAATGTAGCTTCAA  -582
Harosoy       GATTATGTTTTCCCTTTAATTTTTGTGGCCGTTATGGTTTTAACTTTAATGTAGCTTCAA  -588

Nezumisaya    TTATATTCCCAAATGGGTTTTAACTTATTAAAATAGATACATGATTACTACACCAGAGAC  -522
Harosoy       TTATATTCCCAAATGGGTTTTAACTTATTAAAATAGATACATGATTACTACACCAGAGAC  -528

Nezumisaya    ACACACATTACACTACTGAAATGTTAAAATATTATTAAAAATGGGATC--------ATAT  -470
Harosoy       ACACACATTACACTACTGAAATGTTAAAATATTATTAAAAATGGGATCATATATATATAT  -468

Nezumisaya    ATATATATATATATTATAATTGTTTCGAATTAATATGAAATTCTTATCCTGCCCTCATAT  -410
Harosoy       ATATATATATATATTATAATTGTTTCGAATTAATATGAAATTCTTATCCTGCGCTCATAT  -408

Nezumisaya    TATATTTTGCTCCTGCGATACGCTGTTAACTTTTTATTCGTAAAATTTAAAAATATGGTT  -350
Harosoy       TATATTTTGCTCCTGCGATACGTTGTTAAC-TTTTATTCGTAAAATTTAAAAATATGGTT  -349

Nezumisaya    ACGTGTCACCTAAAAATTAGGTGGGACTACATGAAATAATTTCTCCTACAGAAAGTAATA  -290
Harosoy       ACGTGTCACCTAAAAATTAGGTGGGACTACATGAAATAATTTCTCCTACAGAAAGTAATA  -289

Nezumisaya    AATTATATAACTACTTTATTTATTGGGATGATCGACGTATTTATAA-TTTTGTATGAGAA  -231
Harosoy       AATTATATA---ACTTTATTTATTGTGATGATCGACGTGTTTATAATTTTTGTATGAGAA  -232

Nezumisaya    TCACGAGATCACCATGTGCACACTCCTATGTTTACGGTCCACAATTCTACACCACGTAGC  -171
Harosoy       TCACGAGATCACGATGTGCACACTCCTATGTTTACGCTCCACAATTCTACACCACGTAGC  -172

Nezumisaya    CCTCATCAAATCGTTTTCCACCACAAGAACCATAGGATCTCCGTTACTCTCGTTTCTTCT  -111
Harosoy       CCTCATCAAATCGTTTTCCACCACAAGAACCAAAGGATCTCCGTTACTCTCGTTTCTTCT  -112

Nezumisaya    CCAACAACTGAAAGCAAAAACCTTACT-TTCTCACTTATCTCCTTGGCAAAAACAATTTT   -52
Harosoy       -CAACAACTGAAAGCAAAAACCTTACTCTTCTCACTTATCTCCTTGGCAAAAACAATTTT   -53

Nezumisaya    GGTTCAGTTAATTAGTTTCTCCCTTTGCACTTTCTCT-TGTTTCCACAAAGCCATGAAAT     7
Harosoy       GGTTCAGTTAATTAGTTTCTCTCTTTGCACTTTCCCTCGGTTTCCACAAAGCCATGAAAT     7
```
