# Supplementary figures and images for: Linkage mapping, molecular cloning and functional analysis of soybean gene Fg3 encoding flavonol 3-O-glucoside/galactoside (1 → 2) glucosyltransferase
Source: BMC Plant Biol. 2015 May 23;15:126. doi: 10.1186/s12870-015-0504-7 (PMC4494776; doi:10.1186/s12870-015-0504-7)

**A**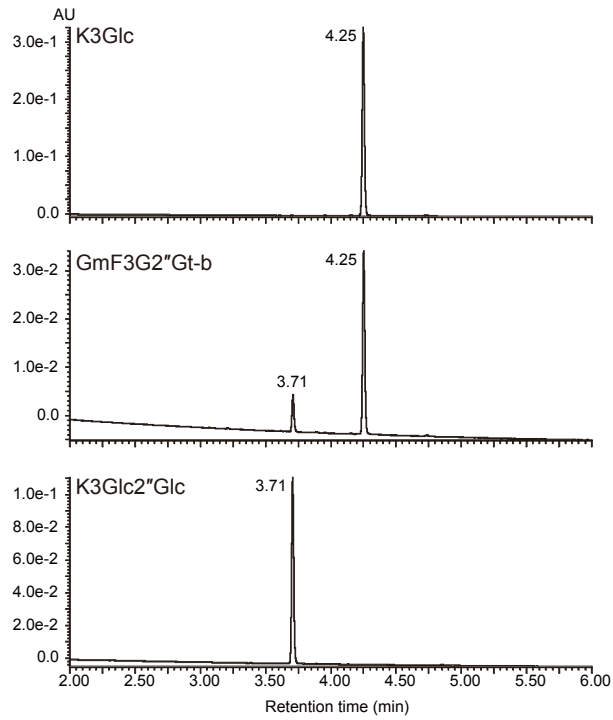**B**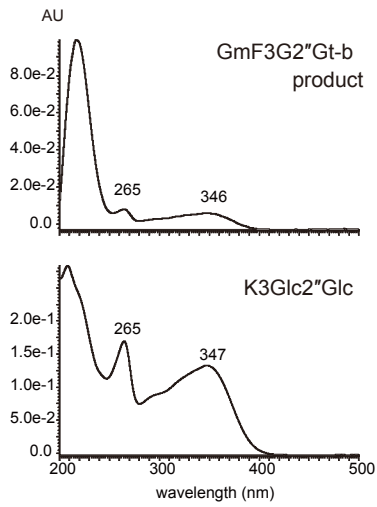**C**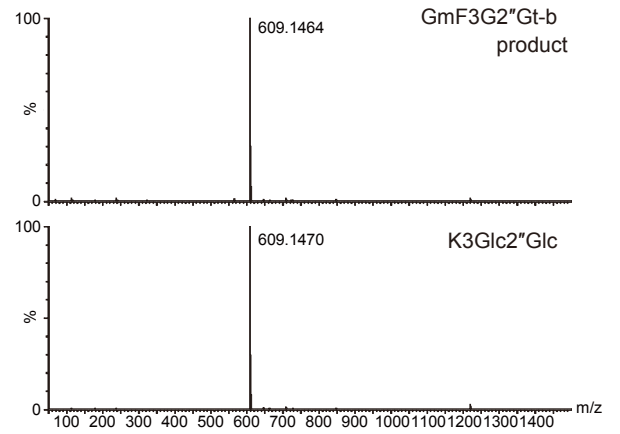**D**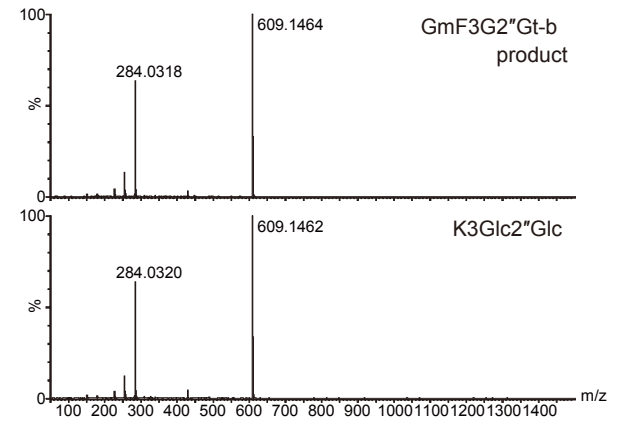

Supplement: Additional file 2: Figure S2. — Identification of reaction product of GmF3G2″Gt-b (cultivar Harosoy). (A) Elution profiles of the standards (kaempferol 3-O-glucoside and kaempferol 3-O-sophoroside) and reaction product of GmF3G2″Gt-b protein. (B) UV spectra of the standard (kaempferol 3-O-sophoroside) and reaction product of GmF3G2″Gt-b protein. Mass spectra (C) and MS/MS spectra (D) of the standard (kaempferol 3-O-sophoroside) and reaction product of GmF3G2″Gt-b protein. K3Glc, kaempferol 3-O-glucoside; K3Glc2″Glc, kaempferol 3-O-sophoroside. [file 12870_2015_504_MOESM2_ESM.pdf]
