# Supplementary material for: Linkage mapping, molecular cloning and functional analysis of soybean gene Fg3 encoding flavonol 3-O-glucoside/galactoside (1 → 2) glucosyltransferase
Source: BMC Plant Biol. 2015 May 23;15:126. doi: 10.1186/s12870-015-0504-7 (PMC4494776; doi:10.1186/s12870-015-0504-7)
Supplement: Additional file 3: Table S1. — Substrate specificity of GmF3G2″Gt in cultivar Harosoy. [file 12870_2015_504_MOESM3_ESM.pdf]

Additional file 3: Table S1. Substrate specificity of *GmF3G2"Gt* in cultivar Harosoy.

	Relative activity (%)
Sugar acceptor[a]	
Kaempferol (Kae) 3-*O*-glucoside[b]	100.0±2.0
Kae 3-*O*-galactoside	89.9±7.9
Kae 3-*O*-rhamnosyl-(1→6)-glucoside	16.1±1.4
Sugar donor[c]	
UDP-glucose	100.0±14.3
UDP-galactose	3.5±0.3
UDP-arabinose	N.D.
UDP-glucuronic acid	N.D.

N.D., not detected.

[a]The reactions were performed with UDP-glucose as the sugar donor.

[b]The product was identified based on comparison with the pertinent standard.

[c]The reactions were performed with kaempferol 3-*O*-glucoside.
