# Supplementary material for: Linkage mapping, molecular cloning and functional analysis of soybean gene Fg3 encoding flavonol 3-O-glucoside/galactoside (1 → 2) glucosyltransferase
Source: BMC Plant Biol. 2015 May 23;15:126. doi: 10.1186/s12870-015-0504-7 (PMC4494776; doi:10.1186/s12870-015-0504-7)
Supplement: Additional file 4: Figure S3. — Multiple alignment of flavonoid glycoside glycosyltransferases (GGTs). The plant secondary product glycosyltransferase motif is underlined. Amino acid residues conserved in flavonoid GGTs are in white font highlighted in black. Amino acid residues conserved in flavonoid G6″GTs and G2″GTs are in white font highlighted in red and blue, respectively. Position of amino acid responsible for sugar specificity in Sg-1 glycosyltransferase is shown by white triangle. Thr residues conserved in G2″GTs are in white font highlighted in green. [file 12870_2015_504_MOESM4_ESM.pdf]

```
CsF7G6"Rt    MHAPSNQHHKMGTESAEADQLHVVMFPWFASGHISPFVQLSNKLSLHGVKVSFFSAPGNIPRIKS--SLN    70
PhA3G6"Rt    MENEMKHSN----------DALHVVMFPFFAFGHISPFVQLANKLSSYGVKVSFFTASGNASRVKS--MLN    61
GmF3G6"Rt    MPSELAMNN----------DELHVVMFPFLAFGHISPFVQLSNKLFSHGVHVTFLSAASNIPRIRS--TLN    61
GmF3G2"Gt    ------MKS----------RPLHIAMYPWLAMGHQIAFLHLCNKLAIRGHKISFITPPKAQAKLEP--FNL    55
AcA3Ga2"Xt   ------MGA----------PTFHIAMYPWFALGHLTPFLHLSNKLARKGHKISFLIPTKTQQKLEP--FNL    55
IpA3G2"Gt    ----MGSQA----------TTYHMAMYPWFGVGHLTGFFRLANKLAGKGHRISFLIPKNTQSKLES--FNL    57
AtF3G2"Gt    -------MG----------SKFHAFMFPWFGFGHMTAFLHLANKLAEKDHKITFLLPKKARKQLES--LNL    54
CmF7G2"Rt    MDTKHQ------------DKPS-ILMLPWLAHGHIAPHLELAKKLSQKNFHIYFCSTPNNLQSFGRNVEKN    58
BpA3G2"Glt   MDSKID------------SKTFRVVMLPWLAYSHISRFLVFAKRLTNHNFHIYICSSQTNMQYLKNNLTSQ    59

CsF7G6"Rt    LTPMADIIPLQIPHVD-GLPPGLDSTSEMTPHMAELLKQALDLMQPQIKTLLSQLKPHFVFFDFTHYWLP   137
PhA3G6"Rt    SAPTTHIVPLTLPHVE-GLPPGAESTAELTPASAELLKVALDLMQPQIKTLLSHLKPHFVLFDFAQEWLP   128
GmF3G6"Rt    LNPAINVISLKFPN--------GITNTAELPPHLAGNLIHALDLTQDQVKSLLLELKPHYVFFDFAQHWLP   122
GmF3G2"Gt    HPNSITFVTINVPHVE-GLPPDAQTTADVTYPLQPQIMTAMDLTKDDIETLLTGLKPDLVFYDFT-HWMP   122
AcA3Ga2"Xt   HPDLITFIPVTVPHVD-GLPLGAETTSDVPYPLQTLLMTAMDRTEKYVEDVLLGLKVDVVFFDFT-HWMP   122
IpA3G2"Gt    HPHLISFVPIVVPSIP-GLPPGAETTSDVPFPSTHLLMEAMDKTQNDIEIILKDLKVDVVFYDFT-HWLP   124
AtF3G2"Gt    FPDCIVFQTLTIPSVD-GLPDGAETTSDIPISLGSFLASAMDRTRIQVKEAVSVGKPDLIFFDFA-HWIP   121
CmF7G2"Rt    FSSSIQLIELQLPNTFPELPSQNQTTKNLPPHLIYTLVGAFEDAKPAFCNILETLKPTLVMYDLFQPWAA   128
BpA3G2"Glt   YSKSIQLIELNLPSSS-ELPLQYHTTHGLPPHLTKTLSDDYQKSGPDFETILIKLNPHLVIYDFNQLWAP   128

CsF7G6"Rt    GLVGSQLGIKTVNFSVFSAISQAYLVVPARKLNN-----SLADLMKSPDGFPATSITSLDEFVARDYLYV   202
PhA3G6"Rt    KMAN-GLGIKTVYYSVVVALSTAFLTCPARVLEPK-KYPSLEDMKKPPLGFPQTSVTSVRTFEARDFLYV   196
GmF3G6"Rt    KLAS-EVGIKSVHFSVYSAISDAYITVPSRFADVEGRNITFEDLKKPPPGYPQNSNISLKAFEAMDFMFL   191
GmF3G2"Gt    ALAK-RLGIKAVHYCTASSVMIGYTLTPARFHQGT--DLMESDLMEPPEGYPDSS-IKLQTHEARVFAAK   188
AcA3Ga2"Xt   SVAK-RLGIKSVNYCIISPATIGYTMSPARQLQGR--ELTEADLMVPPIGYPDFL-IRLRTHEARAFAAR   188
IpA3G2"Gt    SLAR-KIGIKSVFYSTISPLMHGYALSPERRVVGK--QLTEADMMKAPASFPDPS-IKLHAHEARGFTAR   190
AtF3G2"Gt    EIAR-EYGVKSVNFITISAACVAISFVPGR---------SQDDLGSTPPGYPSSK-VLLRGHETNSLSFL   180
CmF7G2"Rt    EAAY-QYDIAAILFLPLSAVACSFL-----------------LHNIVNPSLKYPFFESDYQDRESKNINYF   182
BpA3G2"Glt   EVAS-TLHIPSIQLLSGCVALYALD-----------------AHLYTKP-LDENLAKFPFPEIYPKNR---   182

CsF7G6"Rt    YTKFN-GGPSVYERGIQGVDGCDVLAIKTCNEMEGPYLDFVRTQFKKPVLLTGPLVNPEPPSGELEERWA   271
PhA3G6"Rt    FKSFH-NGPTLYDRIQSGLRGCSAILAKTCSQMEGPYIKYVEAQFNKPVFLIGPVV-PDPPSGKLEEKWA   265
GmF3G6"Rt    FTRFGEKNLTGYERVLQSLGECSFIVFKTCKEIEGPYLDYIETQFRKPVLLSGPLV-PEPSTDVLEEKWS   261
GmF3G2"Gt    RKDTFGSNVLFYDRQFIALNEADLLAYRTCREIEGPYMDYIGKQFNKPVVATGPVILDPPTL-DLEEKFS   257
AcA3Ga2"Xt   RVMKFGGDTRFCDRNFISFSECDAMGFKTCREIEGPYCDYLESQFGKPVLLSGPVIPEPPTS-PLEEIWA   257
IpA3G2"Gt    TVMKFGGDITFFDRIFTAVSESDGLAYSTCREIEGQFCDYIETQFQKPVLLAGPALPVPSKS-TMEQKWS   259
AtF3G2"Gt    S-YPFGDGTSFYERIMIGLKNCDVISIRTCQEMEGKFCDFIENQFQRKVLLTGPMLPEPDNSKPLEDQWR   248
CmF7G2"Rt    LHLTANGTLNKDRFLKAFELSCKFVFIKTSREIESKYLDYFPSLMGNEIIPVGPLIQEPTFKEDD-TKIM   251
BpA3G2"Glt   -DIPKGGSKYIERFVDCMRRSCEIILVRSTMELEGKYIDYLSKTLGKKVLPVGPLVQEASLLQDDHIWIM   246

CsF7G6"Rt    NWLGKFPPKSVIYCSFGSETFLTVDQIKELAIGLEITGLPFFLVLNFPPNVDGQSELVRTLPPGFMDRVK   341
PhA3G6"Rt    TWLNKFEGGTVIYCSFGSETFLTDDQVKELALGLEQTGLPFFLVLNFPANVDVSAELNRALPEGFLERVK   334
GmF3G6"Rt    KWLDGFPAKSVILCSFGSETFLSDYQIKELASGLELTGLPFILVLNFPSNLSAKAELERALPKGYLERVK   330
GmF3G2"Gt    TWLGGFEPGSVVYCCFGSECTLRPNQFLELVLGLELTGMPFLAAVKAPL---GFETVESAMPEGFQERVK   326
AcA3Ga2"Xt   KWLGGFRAGSVIYCAFGSECTLKMNQFQELLLGLVLTGMPFLAVLKPPI---GAKSVEEALPEKFETGVE   326
IpA3G2"Gt    DWLGKFKEGSVIYCAFGSECTLRKDKFQELLWGLELTGMPFFAALKPPF---ETESVEAAIPEELKEKIQ   328
AtF3G2"Gt    QWLSKFDPGSVIYCALGSQIILEKDQFQELCLGMELTGLPFLVAVKPPK---GSSTIQEALPKGFEERVK   318
CmF7G2"Rt    DWLSQKEPRSVVYASFGSEYFPSKDEIHEIASGLLLSEVNFIWAFRLHPD--EKMTIEEALPQGFAEEIE   320
BpA3G2"Glt   KWLDKKEESSVVFVCFGSEYILSDNEIEDIAYGLELSQVSFVWAIRAKT----------SALNGFIDRVG   326

CsF7G6"Rt    --DRGVVHTGWVQQQLILRHESVGCYVCHSGFSSVTEAVISDCQLVLLPLKGDQFLNSKLVAGDLKAGVE   409
PhA3G6"Rt    --DKGIIHSGWVQQQHILAHSSVGCYVCHAGFSSVIEALVNDCQVVMLPQKGDQILNAKLVSGDMEAGVE   402
GmF3G6"Rt    --NRGVVHSGWFQQQLVLKHSSVGCYVCHGGFSSVIEAMVNECQLVLLPFKGDQFFNSKLIANDLKAGVE   398
GmF3G2"Gt    --GRGFVYGGWVQQQLILAHPSVGCFITHCGSGSLSEALVNKCQLVLLPNVGDQILNARMMGTNLEVGVE   391
AcA3Ga2"Xt   --GRGVVHEGWVQQQLILEHPSVGCFITHCGSGSLSEALFNKCQLVLLPNVGDQIINARMMSQNLKVGVE   391
IpA3G2"Gt    --GRGIVHGEWVQQQLFLQHPSVGCFVSHCGWASLSEALVNDCQIVLLPQVGDQIINARIMSVSLKVGVE   393
AtF3G2"Gt    --ARGVVWGGWVQQPLILAHPSIGCFVSHCGFGSMWEALVNDCQIVFIPHLGEQILNTRLMSEELKVSVE   383
CmF7G2"Rt    RNNKGMIVQGWVPQAKILRHGSIGGFLSHCGWGSVVEGMVFGVPIIGVPMAYEQPSNAKVVVDNG-MGMV   388
BpA3G2"Glt   --DKGLVIDKWVPQANILSHSSTGGFISHCGWSSTMESIRYGVPIIAMPMQFDQPYNARLMETVG-AGIE   374

CsF7G6"Rt    VNRRDHDGHFGKEDIFKAVKTVMVDVNKEPGASIRANQKWWREFLLNGQIQDKFIADFVKDLKALA------475
PhA3G6"Rt    INRRDEDGYFGKEDIKEAVEKVMVDVEKEPGKLIRENQKKWKEFLLNKDIQSKYIGNLVNEMTAMAKVSTT-473
GmF3G6"Rt    VNRSDEDGFFHKEDILEALKTVMLEDNKEQGKQIRENHMQWSKFLSNKEIQNKFITDLVAQLKSMA------464
GmF3G2"Gt    VEKGDEDGMYTKESVCKAVSIVMDC-ENETSKRVRANHARIRELLLNKDLESSYVDSFCMRLQEIVEGI---459
AcA3Ga2"Xt   VEKGEEDGLFTGESVCRAVRDAMEE-GSEVAKEVRDNHAKMREFLLNKDLESSYIDNFNKKLQDLLG-----457
IpA3G2"Gt    VEKGEEDGVFSRESVCKAVKAVMDE-KSEIGREVRGNHDKLRGFLMNADLDSKYMDSFNQKLQDLLG-----459
AtF3G2"Gt    VKR-EETGWFSKESLSGAVRSVMDR-DSELGNWARRNHVKWKESLLRHGLMSGYLNKFVEALEKLVQNINLE453
CmF7G2"Rt    VPRDKINQRLGGEEVARVIKHVVLQEEAKQIRRKANEISESMKKIGDAEMS-VVVEKLLQLVKKSE------452
BpA3G2"Glt   VGRDGE-GRLKREEIAAVVRKVVVEDSGESIREKAKELGEIMKKNMEAEVDGIVIENLVKLCEMNN------438
```
